# Surgery

**Published:** 1878-04

**Authors:** 


### Surgery.

Antiseptic Injections into Joints.—Dr. Fr. Rinne (*Centralbl. f. Chirurgie*, 1877, No. 49 and 50). The writer gives an account of 19 cases of the various forms of inflamed knee joints which were treated in this way: The skin of the knee having been thoroughly cleansed and shaved and washed with carbolic acid, while the carbolic spray is playing on it a large well-disinfected trocar is thrust into the joint. As soon as the free motions of the point of the trocar indicate that it has entered the articular cavity the stylet is withdrawn, and the contents of the joint are allowed to flow from the canula. Pressure by the hands upon the sides of the joint often facilitates the withdrawal of the fluid, if it is thick or mixed with clots of fibrine or blood. When the flow ceases, the articular cavity is filled to its utmost capacity with a watery solution of carbolic acid (3 to 5 per cent.). During this process flexion and extension of the joint is practiced, in order to drive the lotion into all the pouches and recesses, and to dislodge what coagula there may be in them. Then the acid is drawn off and the joint filled again; and so the filling is repeated, until the carbolic acid returns perfectly clear and without a trace of pus, blood or fibrine. The canula removed, the punctured wound is dressed with silk protective, and the whole knee covered with Lister's bandage. The leg thus dressed, is put upon a splint and left undisturbed the next 5 or 6 days. But the bandage must not be applied very tightly, for the injections of the acid are expected to produce an acute synovitis, which often is followed by an acute exudation into and a swelling of the joint. Now, if the bandage is tight, it will occasion much pain and necessitate a change of the dressing. The treatment after the sixth day depends entirely on the amount of reaction found in the joint. If there is no exudation or inflammation in the joint, slight pressure is exerted upon it with elastic bandage for two days, and then the patient is allowed to walk with the leg stiffened by a splint. If also then the exudation does not return, moderate movements of the joints are tried; and in two weeks the patient can be discharged cured. But, if on the sixth day, when the bandage is removed, the joint it found distended by acute exudation, rest and compression by elastic bandages must be continued until this reaction has disappeared, to be followed by the same treatment as mentioned before.

By his clinical observations, Dr. R. is lead to believe that the following affections can be cured, or at least benefited, by the antiseptic injections: 1. Acute synovitis, with so large an exudation that the capsule is likely to break. 2. Sub-acute and chronic serous exudation. 3. The mixed forms of exudation—sero-purulent. 4. The traumatic hæmarthrosis.

THORACENTESIS BY ASPIRATION IN ACUTE PLEURISY.—Dieulafoy. (*Lyon Médical*, Dec. 16, 1877.) M. Dieulafoy gives the following *résumé* of the different papers which he has just published in the *Gazette Hebdomadaire.*

Aspiration in acute pleurisy is a simple operation, and absolutely inoffensive; it should be practiced with the needle No. 2, and the quantity of liquid withdrawn at a single sitting, should not exceed a thousand grammes (about a quart).

The accidents attributed to the operation are of different natures, and may be grouped in three classes:

The accidents of the third class, the so-called transformation of a serous liquid into a purulent one, are not imputable to the thoracentesis, for I think I have demonstrated that in that case it is a *natural evolution* of legitimate purulent pleuresy, and in no wise a transformation of the liquid.

The accidents of the second class, syncope, asphyxia, hemiplegia, due to indiginous or migratory clots in the heart or pulmonary vessels, or occurring under the influence of other causes (pleuro-pulmonary gangrene, general state of the subject), should not be placed to the account of the operation, since they are observed in the course of pleurisy before as well as after the thoracentesis, and since they occur from the fact of there being pleuresy, not from the operation.

The accidents of the first class, acute œdema of the lung, pulmonary congestion with or without albuminous expectoration, are the only accidents directly and truly imputable to thoracentesis, but these it is easy to avoid. In collecting all the accidents of this kind together, I have seen that they are always associated with either the immediate issue of a large quantity of fluid, or with complicated pleurisies; hence the precept to limit the quantity of fluid withdrawn at a sitting, and to proportion it to the complications of the pleurisy.

The method of operating being fixed and invariable, there is nothing variable but the indications of the thoracentesis, and these may be stated in two words: The thoracentesis is *urgent* or it is *debatable*. The urgency cannot and should not be based on anything but the estimation of the quantity of liquid effused; in all other cases it is debatable; it is subject to the appreciation of him who follows the course and the nature, an appreciation, so to speak, variable with each new case, for few diseases have such irregular and indecisive turns as accute pleuresy.

